# Heterogeneity in the Response of Different Subtypes of *Drosophila melanogaster* Midgut Cells to Viral Infections

**DOI:** 10.3390/v13112284

**Published:** 2021-11-15

**Authors:** João M. F. Silva, Tatsuya Nagata, Fernando L. Melo, Santiago F. Elena

**Affiliations:** 1 Departamento de Biologia Celular, Universidade de Brasília, Brasília 70910-900, Brazil; joaomarcos.fagundes@gmail.com (J.M.F.S.); tatsuya@unb.br (T.N.); flucasmelo@gmail.com (F.L.M.); 2 Instituto de Biología Integrativa de Sistemas (I2SysBio), CSIC-Universitat de València, 46980 Paterna, València, Spain; 3 The Santa Fe Institute, Santa Fe, NM 87501, USA

**Keywords:** cell-type-specific gene expression, *Drosophila* viruses, dual RNA-seq, single-cell genomics, single-cell RNA-seq, virus-host interaction, antiviral heat shock response

## Abstract

Single-cell RNA sequencing (scRNA-seq) offers the possibility to monitor both host and pathogens transcriptomes at the cellular level. Here, public scRNA-seq datasets from *Drosophila melanogaster* midgut cells were used to compare the differences in replication strategy and cellular responses between two fly picorna-like viruses, Thika virus (TV) and *D. melanogaster* Nora virus (DMelNV). TV exhibited lower levels of viral RNA accumulation but infected a higher number of cells compared to DMelNV. In both cases, viral RNA accumulation varied according to cell subtype. The cellular heat shock response to TV and DMelNV infection was cell-subtype- and virus-specific. Disruption of bottleneck genes at later stages of infection in the systemic response, as well as of translation-related genes in the cellular response to DMelNV in two cell subtypes, may affect the virus replication.

## 1. Introduction

Multi-cellular organisms respond to viral infections at both cellular and systemic levels. How different cell types and how infected and uninfected bystander cells respond to viral infections are questions that are being recently addressed using single-cell RNA-sequencing (scRNA-seq) and other single-cell techniques. As an example, single-cell profiling of Ebola virus (EBOV)-infected immune cells from rhesus macaques revealed that interferon-stimulated genes (ISGs) are down-regulated in infected cells compared to bystanders [1], shedding light into previous seemingly contradictory results from studies of EBOV infection in culture and in vivo, where ISGs and downstream signaling genes were, respectively, down- and up-regulated compared to their healthy counterparts [2,3,4,5,6,7]. Similarly, studies using scRNA-seq found that bystander cells from mice infected with influenza virus A (IAV), and bystander cells from patients positive for severe acute respiratory syndrome coronavirus-2 (SARS-CoV-2) show an over-expression of ISGs compared to cells from healthy individuals [8,9], stressing the importance of a systemic response to these respiratory viruses. Yet, this powerful technique has been largely applied to viral infections in mammalian cells, and studies in non-mammalian hosts are currently missing.

The fruit fly (*Drosophila melanogaster* Meigen) is an attractive invertebrate model for studying virus-host interactions [10] in which RNA interference (RNAi) plays a major antiviral role [11,12]. Heat shock response to viral infections has also been shown to contribute to antiviral defense by restricting viral replication [13]. Overexpression of the heat shock factor (Hsf) and Hsp70 induce resistance to viral infections in transgenic flies. In particular, overexpression of Hsf diminished viral loads to undetectable levels in some instances, suggesting that these transformed flies were cleared from viral infections [13].

Novel sequencing technologies are allowing the discovery of novel RNA viruses in both wild and stock flies [14] that may serve as models for studying virus-host interactions. However, the biology of only a small subset of these viruses has been investigated in-depth, such as *D. melanogaster* sigma virus (DMelSV; genus *Sigmavirus*, family *Rhabdoviridae*), Drosophila C virus (DCV; genus *Cripavirus*, family *Dicistroviridae*), and, more recently, *D. melanogaster* Nora virus (DMelNV; unclassified picorna-like virus), an enteric virus that is known to cause persistent infections on both wild and stock flies with no obvious pathological outcome [11,14,15,16].

**Figure 1:**
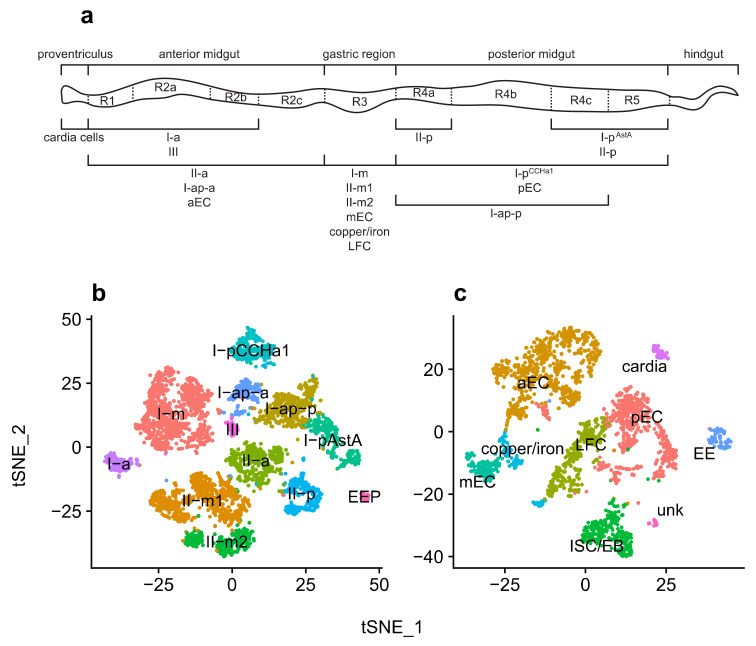
Spatial distribution of EE cells and of viral infections. (**a**) Schematic representation of the fly gastrointestinal tract showing the spatial distribution of EEs (adapted from [22]; published under creative commons license). (**b**) t-SNE (*t*-distributed stochastic neighbor embedding) reduction plots of EEs with identified clusters. (**c**) t-SNE reduction plots of cells from the entire fly midgut with identified clusters. In both t-SNE plots, each point represents a single cell, and colors indicate labeled cell types.

The gastrointestinal tract of the fruit fly is composed of diverse cell types that perform essential tasks, such as nutrient uptake and secretion of neuropeptide hormones. Enterocytes (ECs) are responsible for the secretion of digestive enzymes and nutrient uptake. These cells possess specialized gene expression profiles depending on regionalization and can be divided into ECs from the anterior (aEC), middle (mEC), and posterior (pEC) portions of the fly’s midgut as well as into a few subtypes [17]. The middle region, also known as gastric region, of the fly’s midgut, resembles the mammalian stomach due to acidification by copper cells [18]. Enteroendocrine cells (EEs) secrete a variety of neuropeptide hormones that play an important role in controlling many physiological processes. They are scattered throughout the gastrointestinal tract and produce more than 20 neuropeptide hormones, including allatostatines A, B, and C (AstA, AstB, and AstC, respectively); tachykinin (Tk); neuropeptide F (NPF); diuretic hormone 31 (DH31); and CCHamide-1 and -2 (CCHa1 and CCHa2, respectively) [19,20,21,22]. Recently, ten EE subtypes were identified in the fruit fly. These subtypes can be divided into two major classes: class I is composed of cells expressing AstC, and class II is composed of cells that express Tk [22,23]. In addition to these classes, a subtype dubbed III that does not express neither AstC nor Tk has been also identified. Further classification of EE subtypes is based on whether they are located in the anterior, medium (gastric region), or posterior regions of the gastrointestinal tract (-a, -m, and -p suffixes; Figure 1a) and on their gene expression [23].

Here, publicly available Drosophila scRNA-seq datasets from the midgut epithelium cells were used to investigate the infection dynamics of two viruses, DMelNV and the recently discovered Thika virus (TV; unclassified picorna-like virus). By analyzing both viral RNA accumulation and host transcription levels in a manner that is analogous to dual RNA-seq [24], we show that in the presence of multiple infections, it is possible to use scRNA-seq to analyze the transcription of the host and multiple pathogens simultaneously. Not only we have been able to monitor both virus replication and host transcriptional response to infection, we have been also able to simultaneously compare the replication strategies of two viruses and some of the cellular responses to these viruses in vivo, revealing more of the uniqueness and similarities of viral infections at the single-cell level and providing possible new models for invertebrate viruses.

## 2. Materials and Methods

### 2.1. Data Collection

Raw sequencing data were downloaded from NCBI SRA database (https://www.ncbi.nlm.nih.gov/sra last accessed 15 November 2021; BioProject accession: PRJNA547484). Briefly, these data were generated by following 10× Genomics GemCode protocol [25] using ~8000 EEs harvested from the midgut of ~200 female fruit flies (CG32547-GAL4 > GFP line) aged between five and seven days [23]. GFP was used to sort cells with a FACS Aria III sorter (BD Biosciences,) and sequencing was performed at an Illumina X10 platform. Additionally, a fruit fly scRNA-seq dataset from the entire midgut, hereafter referred to as midgut atlas (MA) dataset, was also obtained through SRA (BioProject accession: PRJNA493298). Only data generated by 10x technology were analyzed. Briefly, guts from seven days old female flies (esg-sfGFP/+, pros-GAL4 > RFP/+ line) were dissected, and libraries were prepared following 10x Genomics GemCode protocol [17]. Two technical replicates for two samples were prepared for this data, resulting in a total of four libraries.

### 2.2. Identification of Viruses in scRNA-Seq Datasets

For each dataset, the FASTQ files corresponding to the transcripts were trimmed with BBDuk v38.87 (BBMap package; sourceforge.net/projects/bbmap/; last accessed 15 November 2021), with parameters ktrim = r ref = adapters k = 21 qtrim = r trimq = 10. Trimmed reads were concatenated and aligned to the *D. melanogaster* reference genome (accession GCA_000001215.4) with BWA v0.7.15 [26] using default parameters. Next, mapped reads were filtered out with samtools v1.12 [27]. The remaining reads were assembled with MEGAHIT v1.2.9 [28], and the resulting contigs were queried against the nr database (download in June 2021) with DIAMOND BLASTX [29]. False positives were determined based on further BLAST searches. Three picorna-like viruses were found in the EE dataset: DCV, DMelNV, and TV; and two viruses were found in the MA dataset: DMelNV and Drosophila A virus (DAV).

### 2.3. Filtering and Cluster Generation

Barcode processing and gene counts were performed with CellRanger v3.1.0 (10× Genomics, CA, USA) using an edited *D. melanogaster* reference genome that included the viral sequences from DCV (accession AF014388), DMelNV (accession GQ257737), and TV (accession KP714072). Downstream analysis was performed with Seurat v3.1.4 [30]. A total of 4994 cells containing between 200 and 3000 detected genes and <5% mitochondrial genes were retained. Scaling and normalization were performed with the SCTransform function [31], with mitochondrial genes and virus counts regressed out. Principal component analysis (PCA) and *t*-distributed stochastic neighbor embedding (t-SNE) reductions were performed with top 20 PCs, and clusters were generated at a 0.4 resolution. Clusters were identified using marker genes [23], as shown in Supplementary Figure S1a.

The same analysis was performed for the MA dataset, albeit with some minor differences. The DAV genome (accession FJ150422) was added to the GTF and FASTA files; however, no counts from this virus were found in cells after quality filtering. Possibly, the lack of a poly(A) tail hampered the accurate detection of this virus in this dataset. CellRanger v3.1.0 was run separately for each library. Technical replicates counts were merged, and the two samples were integrated using SCTransform normalized counts with FindIntegrationAnchors and IntegrateData functions in Seurat v3.1.4. After filtering, 2375 cells containing between 200 and 3000 detected genes and <25% mitochondrial genes remained. All integration steps, PCA, and t-SNE reduction were performed with top 50 PCs, and clusters were generated at a 1.0 resolution. Clusters were identified with a set of marker genes [17], as shown in Supplementary Figure S1b.

After identification of cell types in these datasets, ambient RNA contamination was removed with CellBender [32]. Ambient RNA contamination is ubiquitous in droplet-based scRNA-seq protocols, and filtering off background RNA contamination can ameliorate downstream analysis, such as differential expression [32]. This analysis was performed on each library independently. CellBender was run on the raw output count matrices produced by CellRanger in which viral counts were removed.

### 2.4. Determination of Infected Cells

Cells were determined to be infected based on the estimated fraction of ambient RNA in each cell and the probability of finding viral unique molecular identifiers (UMI) in empty droplets. The profile of ambient RNA estimated from cell-free empty droplets is a compelling approach to call infected cells since it accounts for the possible contamination of viral particles in cell-containing droplets [1]. First, for each cell *i*, we estimated the fraction of ambient RNA, *A*(*i*), by dividing the number of UMIs filtered out with CellBender by the total number of non-viral UMIs before the removal of background RNA. Next, the probability of finding viral UMIs in ambient RNA, *V*(*a*), was calculated from the proportion of viral UMIs in empty droplets. Cell barcodes not called by CellRanger containing <100 non-viral UMIs were considered empty droplets. Then, for each cell *i*, the probability of infection *P*(*i*) was calculated based on the following Binomial survival, or reliability, function with *N* trials and probability of success *p*:

*P*(*i*) = *S*[*V*(*i*) | *p*, *N*] = 1 − *F*[*V*(*i*) | *p* = *A*(*i*)*V*(*a*), *N* = *UMI*(*i*)], (1)

where *F*[·] is the Binomial CDF. *V*(*i*) and *UMI*(*i*) are, respectively, the number of viral and total UMIs in cell *i,* and *p* is the probability of finding *V*(*a*) viral UMIs derived from ambient RNA contamination *A*(*i*). Cells with *P*(*i*) < 0.01 were determined to be infected. In addition, a second criterion in which infected cells need to have at least two viral UMIs was applied. Notably, no TV-derived reads were found in the ambient RNA, and cells were determined to be infected based solely on the second criterion.

### 2.5. Virus Infection/Replication Analyses

The proportion of viral counts in each cell was multiplied by a factor of 10,000 and log(pseudocount + 1)-transformed. One-way ANOVA tests were performed to investigate whether the accumulation of DMelNV and TV are influenced by cell subtype using R v4.0.3. Significant results were further investigated by performing Tukey–Kramer post-hoc tests using the agricolae R package v1.3-2 (https://CRAN.R-project.org/package=agricolae; last accessed 15 November 2021) in R v4.0.3.

### 2.6. Gene Expression Analyses

Differential gene expression analysis was performed with glmGamPoi [33]. A one-way layout with one level for each cell subtype/infection status was used. An intercept term was not included in this analysis. Only uninfected cells with no viral UMIs were included as uninfected cells, and the unk cell type from the MA dataset was not included in this analysis. Generic cellular response differentially expressed genes (DEGs) were identified by testing for the effect of infection in all cell subtypes. The generic response to DMelNV was tested for the EE and MA datasets separately. Cell-subtype-specific DEGs were identified by testing for the effect of the interaction between infection and cell subtype for each subtype separately. For TV, the response to cells located in the posterior region of the midgut was also tested with the corresponding contrast. For each virus, cell-subtype-specific response DEGs were pulled together in lists of up- and down-regulated genes. To find genes which expression correlates to viral accumulation, we ran glmGamPoi with expression matrices containing only infected cells using cell subtype as a factor and the percentage of viral UMIs as a covariate, and then, tests were conducted for DEGs by setting only the percentage of viral UMIs as a contrast. For every list of up- or down-regulated DEGs composed by at least three genes, pathway enrichment analysis was conducted with ReactomePA [34].

### 2.7. Gene Regulatory Network Activity Analysis

SCENIC [35] R package v1.2.4 was used to infer regulon (a regulatory network composed of a transcription factor and its target genes) activity on both EE and MA datasets separately. CellBender-corrected counts were used as input to this analysis. Genes expressed in at least 1% of the cells and having at least 3 × total number of cells × 0.01 (which corresponds to the amount of UMI counts a gene would have if it had 3 counts in 1% of the cells) were retained. Network inference based on co-expression was performed with GENIE3 [36], and the area under the curve (AUC) activity values for each regulon was then obtained with AUCell. Differential regulon activity of the AUC values was conducted via the Seurat FindAllMarkers function with the default Mann–Whitney U-test (Supplementary File S3).

### 2.8. Gene Network Analyses

A high quality predicted interactome of *D. melanogaster* was downloaded from http://drosophila.biomedtzc.cn v2018_01 (last accessed 15 November 2021) [37]. Gene interaction networks were analyzed as undirected graphs with the igraph R package v1.2.5 [38]. The degree probability distribution and the betweenness centrality of each node was computed for the interactome network. Then, a linear regression on the degree probability distribution in the log-log space was computed to obtain the critical exponent, γ, of the power-law fit. For each list of DEGs containing more than three genes, the corresponding subnetwork was extracted from the complete interactome, and its critical exponent was obtained as described above. Next, we performed *t*-tests between the critical exponent of the full interactome and each subnetwork to test for significant differences in the degree distribution between the subnetwork and complete interactome. The betweenness centrality of DEGs were computed using the complete network. One-tailed Man–Whitney U-tests were then performed to compare the betweenness centrality of the DEGs with that obtained from all genes in the network, considering the upper tail of the distribution. All *p*-values were adjusted by the Benjamini–Hochberg method. Articulation points were determined with the articulation_points function.

### 2.9. DMelNV Infection Bulk RNA-Seq Data Analysis

A list of DEGs from DMelNV-infected female flies was obtained from Lopez et al. [39]. These data contains DEGs detected at 2, 10, 20, and 30 days post-eclosion. Briefly, these data were generated by stablishing DMelNV-infected white-eyed flies (w^1118^; Vienna Drosophila Resource Center, Vienna, Austria) stocks via fecal-oral infection. RNA extraction was performed with TRIzol reagent (ThermoFisher Scientific, Waltham, MA, USA) on triplicates for each time point, and samples were sequenced at an Illumina HiSeq system platform. Fragments per kilobase of transcript per million mapped reads (FPKM) values were used to determine DEGs. Gene network analysis was performed for up- and down-regulated genes as described above for each time point separately.

### 2.10. Analysis of the Effects of Infection on the I-p^AstA^ Subtype

Cluster generation analysis of the I-p^AstA^ subtype was done as described previously with some minor differences. First, normalization was performed on CellBender-generated counts based on cell size with the NormalizeData function. The top 20 PCs were used for cluster generation and t-SNE reductions, and clusters were generated at a 0.5 resolution.

## 3. Results

### 3.1. Detection of RNA Viruses in Public D. melanogaster scRNA-Seq Datasets

A publicly available EE scRNA-seq dataset from *D. melanogaster* (BioProject accession: PRJNA547484) was obtained from the Sequence Read Archive (SRA; https://www.ncbi.nlm.nih.gov/sra; last accessed 15 November 2021) and investigated for the presence of viruses. A total of 2,672,571 out of 166,308,891 reads (1.6%) remained after filtering Drosophila-derived reads. Forty contigs were assembled and queried against the non-redundant (nr) GenBank database, leading to the identification of three RNA viruses in this dataset: DCV, DMelNV, and TV (Table 1). We also identified one contig with similarity to the drosophila endosymbiont *Wolbachia pipientis*, indicating that some flies might be infected by this bacterium. Hits to *Saccharomyces cerevisiae* and *Muntiacus reevesi* are most likely false positives or derived from sample contamination. Next, reads were aligned to the *D. melanogaster* and virus genomes to obtain gene and viral counts for each cell, which were used for clustering purposes. All clusters were identified based on a set of marker genes [23] (Supplementary Figure S1a). In total, 6, 55, and 766 cells infected with DCV, DMelNV, and TV, respectively, were found. Figure 1a shows a schematic representation of *D. melanogaster* gastrointestinal tract, along with the approximate spatial distribution of EEs subtypes. t-SNE reduction plots of the cell clusters are shown in Figure 1b. No infected EE progenitor (EEP) could be found, and no cell from the I-a and III subtypes was found to be infected with DMelNV. Only eight cells were coinfected with DMelNV and TV, which is in perfect agreement with the hypothesis of independent infection based on the proportion of cells singly infected with DMelNV and TV (probabilities of infection with DMelNV 0.0110 and with TV 0.1534; hence, the probability of coinfection is 0.0017 and the expected number of coinfected cells 0.0017 × 4994 = 8.4898; Binomial test *p* = 0.4902).

In addition to the EE scRNA-seq dataset, a publicly available scRNA-seq dataset from the entire midgut of fruit flies was also investigated for the presence of viruses (BioProject accession: PRJNA493298). In these data, to which we will refer as midgut atlas (MA) dataset, 173,810,419 out of 467,222,107 reads (37.2%) were retained after filtering. Queries of 60 contigs against the nr database led to the detection of DMelNV and DAV, which is likely a non-polyadenylated virus [40] (Table 1). However, no counts from DAV were found in cells after quality control, suggesting contamination from viral particles. Likely, the lack of a poly(A) tail hindered a precise detection of DAV in this dataset. Further inspection of one contig with hit to *D. melanogaster* tetravirus SW-2009a revealed that it consists of a false positive. Contigs with hits to *Muntiacus reevesi*, *S. cerevisae,* and *Staphylococcus aureus* are likely false positives or are derived from sample contamination. Like the EE dataset, cell clusters were generated and identified based on a set of marker genes [17] (Supplementary Figure S1b). Figure 1c shows t-SNE plots of major cell types that were annotated in this dataset. Clusters composed of aECs, mECs, and pECs were identified, along with clusters composed by cardia cells, located in the proventriculus region; intestinal stem cells and enteroblasts (ISC/EBs); large flat cells (LFCs), which are located in the posterior gastric region; copper/iron cells, located in the gastric region; and one cluster composed by unknown cell types (hereafter referred as “unk”). EEs were also identified in this dataset; however, their subtypes could not be reliably annotated. Clusters composed by differentiating ECs and EC-like cells [17] were not identified, possibly due to removal of these cells in our pre-processing step and/or due to them clustering to their most similar ECs. A higher prevalence of DMelNV was noted in this dataset, with 131 cells infected with this virus. However, some cell types had only a few DMelNV-infected cells, such as EEs (4), cardia (2), and unk (1), which likely hampered downstream analysis in these cell types.

### 3.2. DMelNV and TV Exhibit Different Patterns of Replication

**Figure 2:**
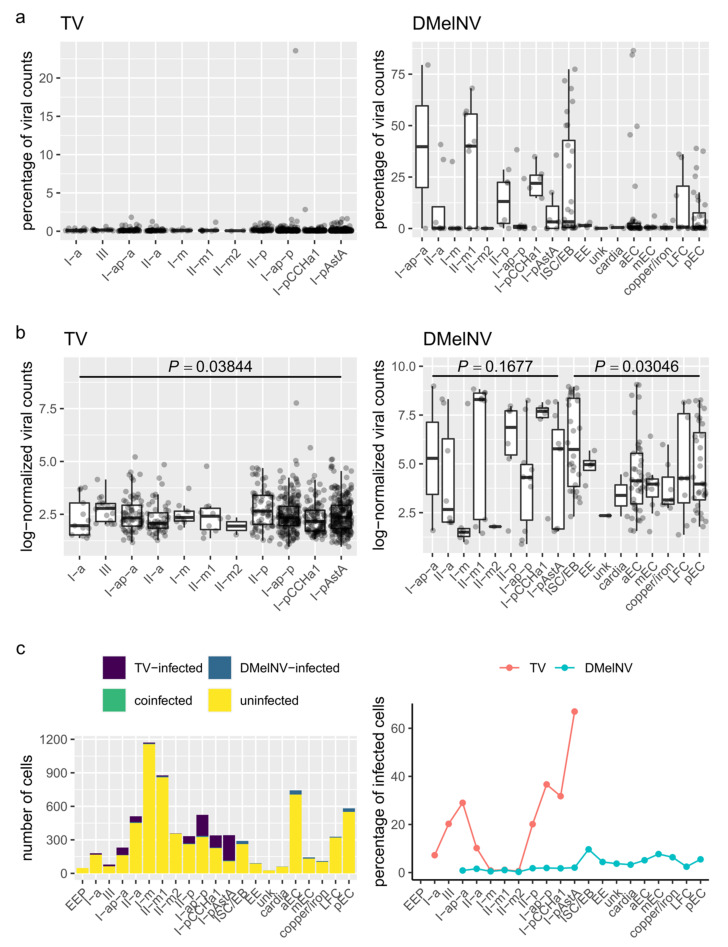
Statistic describing the spatial heterogeneity in the infection of TV and DMelNV along *D. melanogaster* gastrointestinal tract. (**a**) Boxplots showing the percentage of viral RNA from TV and DMelNV for each cell subtype. Each point represents an individual cell. (**b**) Boxplots showing log-expression values of TV and DMelNV for each cell subtype. Each point represents an individual cell. (**c**) Number (left) and percentage (right) of cells from each subtype infected with TV and DMelNV.

The replication level of both DMelNV and TV was evaluated by analyzing both the percentage of viral RNA in the cells and log-normalized expression values (Figure 2a,b). A dramatic contrast in the replication levels between the two viruses was found. TV infected a higher number of cells compared to DMelNV but exhibited lower replication levels per cell. The percentage of viral RNA in TV-infected cells was always below 5% with only one exception (a single-cell I-ap-p), whereas for DMelNV, the percentage of viral RNA was up to ~80% of total mRNA. The percentage of infected cells on each cluster also varied drastically between these viruses (Figure 2c). TV infected a very low proportion of cells from subtypes located in the gastric region and was found in 66% of the cells of the I-p^AstA^ cell subtype located in the posterior region of the gastrointestinal tract that is characterized by the expression of AstA, AstC, and CCHa1.

One-way ANOVA tests showed a significant influence of EE subtype on the expression level of TV (*F*_10,755_ = 1.9289, *p* = 0.0384) but not of DMelNV (*F*_8,46_ = 1.5465, *p* = 0.1677) although a significant influence of cell type was found for DMelNV when analyzing the MA dataset (*F*_8,122_ = 2.2179, *p* = 0.03046), as shown in Figure 2b.

### 3.3. Generic and Cell-Subtype-Specific Transcriptional Response to TV and DMelNV

**Figure 3:**
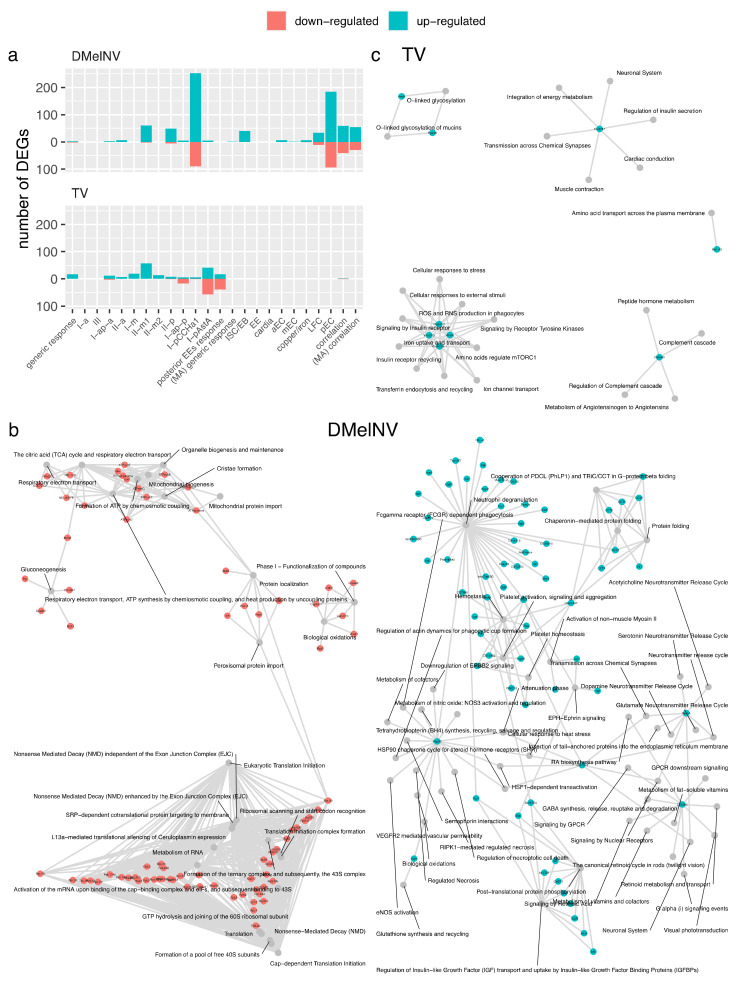
Differential expression and pathway enrichment analyses. (**a**) Number of differentially expressed genes for each generic and cell-subtype-specific test. (**b**,**c**) Summary of all enriched pathways and its related genes found in the cellular response to TV and DMelNV infection, respectively. Each pathway is represented by a grey node connected to genes found to be differentially expressed.

Differential expression (DE) analyses were conducted to uncover the generic and cell-subtype-specific cellular responses to TV and DMelNV (Supplementary File S1). Seventeen, four, and one differentially expressed genes (DEGs) were found in the generic response to TV and DMelNV on the EE dataset and to DMelNV on the MA dataset, respectively (Figure 3a). Notably, in the generic response to TV, all DEGs were up-regulated. The generic response to TV of subtypes from the midgut posterior region was also investigated, with 17 and 39 genes found to be up- and down-regulated, respectively. Pathway enrichment analyses were not significant for these small subsets of genes, with the exception of up-regulated genes in response to DMelNV on the EE dataset. In this list, genes related to regulation of heat shock factor 1 (HSF1)-mediated heat shock response, cellular response to stress and heat stress, and GABA synthesis, among a few others, were enriched (Figure 3b; Supplementary File S2). Despite the fact that only eight cells coinfected with DMelNV and TV were annotated, we were able to detect an up-regulation of Hsc70-3 and a down-regulation of the neuroendocrine protein 7B2 in these cells (Supplementary File S1).

When testing for cell-subtype-specific responses, the number of detected DEGs varied greatly between cell subtypes, with a few instances where no DEGs were found (Figure 3a; Supplementary File S1). The highest number of detected DEGs was in the DMelNV-infected I-p^CCHa1^ and pEC subtypes. Up-regulated cell-subtype-specific DEGs in response to TV infection were enriched in genes related to glycosylation, insulin receptor recycling and insulin secretion, and cellular response to stress as well as in genes related to the immune system, such as complement cascade, and ROS and RNS production in phagocytes, in addition to others (Figure 3c; Supplementary File S2). It is worth noticing that these insulin- and immune-related reactome pathways in Drosophila were inferred based on human orthologs (see https://reactome.org/documentation/inferred-events; last accessed 15 November 2021) [41], and as such, genes in these categories were manually inspected. These pathways were enriched due to the up-regulation of the vacuolar H^+^ ATPses subunits SFD, 55, 39-1, and 68-2 (VhaSFD, Vha55, Vha39-1, and Vha68-2, respectively), which are multirole proton pumps often expressed in a region-specific manner in the fruit fly’s midgut [42,43]. Up-regulated cell-subtype-specific DEGs in DMelNV-infected cells showed an enrichment of genes related to protein folding and cellular response to heat stress and neutrophil degranulation, among others. Down-regulated cell-type-specific DEGs in response to DMelNV showed an enrichment of genes mostly related to translation, nonsense-mediated decay, gluconeogenesis, and respiratory chain, among others (Figure 3b; Supplementary File S2).

In addition to testing for the effect of infection and the interaction between infection and cell subtype in DE analysis, the effect of viral percentage on each infected cell was also tested to find genes whose expression correlates to viral RNA accumulation. As viral RNA accumulation is expected to increase with time, we hypothesized that these genes may serve as indicatives of the time a cell has been infected. The expression of 3, 100, and 84 genes were found to be correlated to the accumulation of TV and DMelNV on the EE dataset and of DMelNV on the MA dataset, respectively. Possibly, the narrow range of variation of the accumulation level of TV hampered the detection of host-correlated genes. Henceforth, only genes in which expression values were correlated to the accumulation of DMelNV were further analyzed. Pathway enrichment analyses results for these genes were similar to the results obtained above with lists of DEGs. Pathways related to protein folding, response to heat stress, retinoid metabolism, and transport and glutathione synthesis and recycling were enriched in genes that correlated positively to DMelNV accumulation on the EE dataset. In both EE and MA datasets, an enrichment of pathways mostly related to respiratory chain was found for genes that correlated negatively to DMelNV accumulation. Pathways related to translation were also found for genes that correlated negatively to DMelNV accumulation on the MA dataset only (Supplementary File S2).

#### 3.3.1. Heat Shock Response to Infection Is Cell-Subtype- and Virus-Specific

**Figure 4:**
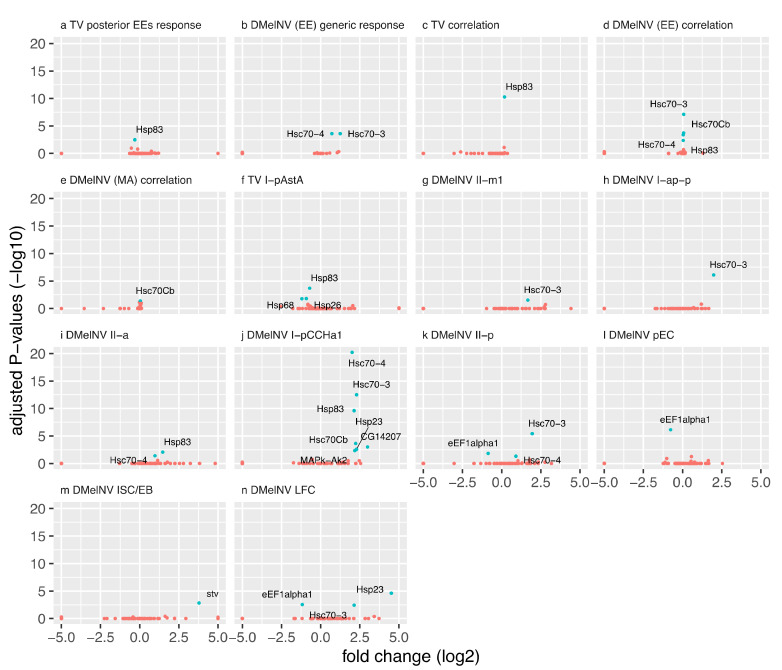
(**a**–**n**) Volcano plots of genes associated with heat shock response. Each panel correspond to a differential expression test where at least one gene associated with cellular response to heat stress (Reactome pathway: R-DME-3371556) was differentially expressed (adjusted *p* < 0.05). These genes are highlighted in blue. Log_2_-fold changes of genes that expression correlate to viral RNA accumulation correspond to the expected increase in gene expression when the viral percentage increases by one unit.

We focused on analyzing the heat shock response to DMelNV and TV given its well-known role in antiviral defenses [13] and the enrichment of heat stress-related pathways in lists of DEGs in response to DMelNV infection. Differential expression of genes associated with cellular response to heat stress (Reactome pathway: R-DME-3371556) was detected in one and eight cell subtypes as a response to TV and DMelNV infection, respectively (Figure 4). In EEs, Hsc70-3 and -4 were up-regulated as a generic response to DMelNV, and the expression of four heat shock proteins correlated positively to DMelNV accumulation (Figure 4b,d,e). Nevertheless, the number of differentially expressed heat shock proteins varied substantially between EE subtypes, where seven genes associated with heat stress were found to be up-regulated in the I-p^CCHa1^ subtype (Figure 4j). In the MA dataset, fewer differentially expressed heat stress-associated genes were detected. The expression of Hsc70Cb was found to correlate positively to DMelNV accumulation in this dataset, while Hsc70-3, Hsp23, starvin, and the elongation factor eEF1a1 were differentially expressed in a cell-subtype-dependent manner (Figure 4). In mammalian cells, the eEF1a1 homolog recruits HSF1 to induce a heat shock response [44]. Therefore, its down-regulation in the II-p, pEC, and LFC cell types may be associated with a less robust antiviral defense. The differences between the EE and MA datasets might reflect differences in the fruit fly’s genotype from each experiment. Regardless, these results suggest that heat shock response to DMelNV is cell-subtype-specific.

Interestingly, heat shock proteins were down-regulated in response to TV (Figure 4a,f). Hsp83 was down-regulated in TV-infected EEs subtypes from the posterior region of the fly’s midgut, but surprisingly, its expression correlated positively to TV RNA accumulation (Figure 4a,c). Proteins Hsp26, Hsp83, and Hsp68 were down-regulated in the I-p^AstA^ subtype in response to TV infection (Figure 4f). Down-regulation of eEF1a1 and heat shock proteins in response to DMelNV and TV infection, respectively, may indicate that these viruses employ different strategies to target and suppress antiviral heat shock response.

#### 3.3.2. Hsf Regulon Activity Is Higher in EEs and Variable between Cell Subtypes

**Figure 5:**
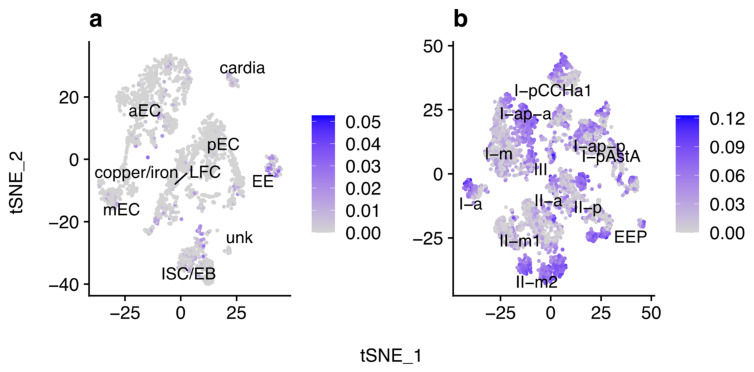
Hsf regulon activity in cells from the entire fly’s midgut (**a**) and EEs (**b**) showing AUC values for each cell. Each point represents a single cell.

The activity of the Hsf regulon was investigated to explore whether heat shock response varies according to cell subtype. By analyzing cells from the whole midgut, we found that Hsf activity is higher in EEs (Figure 5A; Mann–Whitney *U*-test; adjusted *p* < 0.0001; average log_2_-fold change = 0.0133). Interestingly, Hsf activity was highly variable both between and within EEs subtypes (Figure 5b). Higher levels of Hsf regulon activity between and within cell types/subtypes may translate to higher intrinsic antiviral immunity, and may at least partially explain heterogenic response to viral infections.

### 3.4. Time-Course Analysis of the Systemic Response to DMelNV

**Figure 6:**
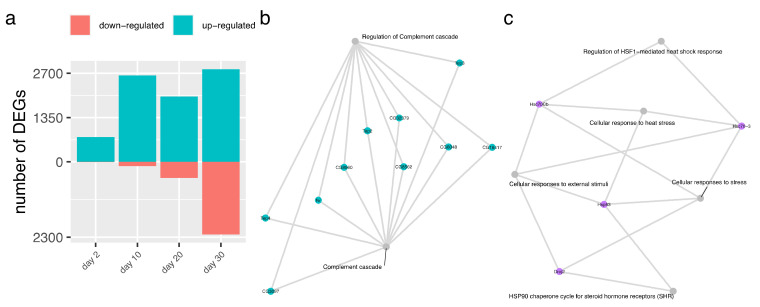
Time course analysis of the systemic response to DMelNV. (**a**) Number of differentially expressed genes at each time point. (**b**) Enriched pathways related to immune defense in up-regulated genes at 20 and 30 days post-eclosion. (**c**) Enriched pathways in genes which expression correlate to DMelNV RNA accumulation but are down-regulated at 20 and/or 30 days post-eclosion. Each pathway is represented by a grey node connected to genes found to be differentially expressed.

Bulk RNA-seq data from DMelNV-infected flies [39] were obtained to analyze the systemic response to this virus. Most DEGs were found to be up-regulated, especially at earlier time points (Figure 6a). An increase in the expression of immune genes overtime has been previously found in this dataset [39]. Accordingly, an enrichment of genes related to complement cascade was found in up-regulated genes only at 20 and 30 days post-eclosion (Figure 6b). Inspection of these genes revealed the presence of known Drosophila immune-related genes, such as thioester-containing protein 2, 3, and 4 (Tep2, 3, and 4, respectively) [45]. The *Aedes aegypti* Teps (*Ae*Teps), in particular *Ae*Tep1 and *Ae*Tep 2, were shown to regulate flavivirus infection [46]. A total of 270 genes were present in both cellular and systemic response to DMelNV, in addition to 68 genes whose expression was correlated to DMelNV accumulation that were also present in the systemic response to this virus at any time point (Supplementary File S1). Up- and down-regulated genes in the cellular and systemic responses were generally consistent although in some cases, genes that were up-regulated in the cellular response were down-regulated in the systemic response and vice versa. The most interesting cases were from genes that were down-regulated only at later stages of infection (20 and 30 days post-eclosion) in the systemic response to DMelNV, but their expression was positively correlated to the virus’ RNA accumulation in the cellular response (15 genes) or were up-regulated in the cellular response (84 genes). An enrichment of genes related to cellular response to stress, heat stress and external stimuli, as well as related to HSP90 chaperone cycle for steroid hormone receptors and regulation of HSF1-mediated heat shock response was found in genes whose expression correlated positively to DMelNV accumulation but are down-regulated in the systemic response at 20 or 30 days post-eclosion (Figure 6c).

### 3.5. Mapping DEGs into D. melanogaster Interactome

A network biology approach was taken to study the effects of the DEGs on the host transcriptome. In this approach, the *D. melanogaster* interactome is represented by a network where genes (nodes) are connected to each other by edges to represent their coordinated expression. Topological parameters of a predicted *D. melanogaster* interactome network were computed to investigate whether DEGs constitute essential nodes in the network. Enrichment of hubs and bottlenecks in lists of DEGs were investigated by computing two parameters: degree, i.e., the number of interactions of a gene and betweenness centrality, i.e., the number of shortest paths that pass through a gene in the network. Essential genes are very likely defined by either a high degree (hubs) or high betweenness centrality (bottlenecks) [47,48]. We also sought to determine articulation points in the fruit fly’s interactome, which are nodes that increase the number of connected components in a graph when they are removed, essentially disconnecting the network. The values of the critical exponent, mean degree, and mean betweenness centrality for each DEG list as well as a list of all articulation points are shown in Supplementary File S2.

#### Regulation of Essential Genes in Response to DMelNV and TV

**Figure 7:**
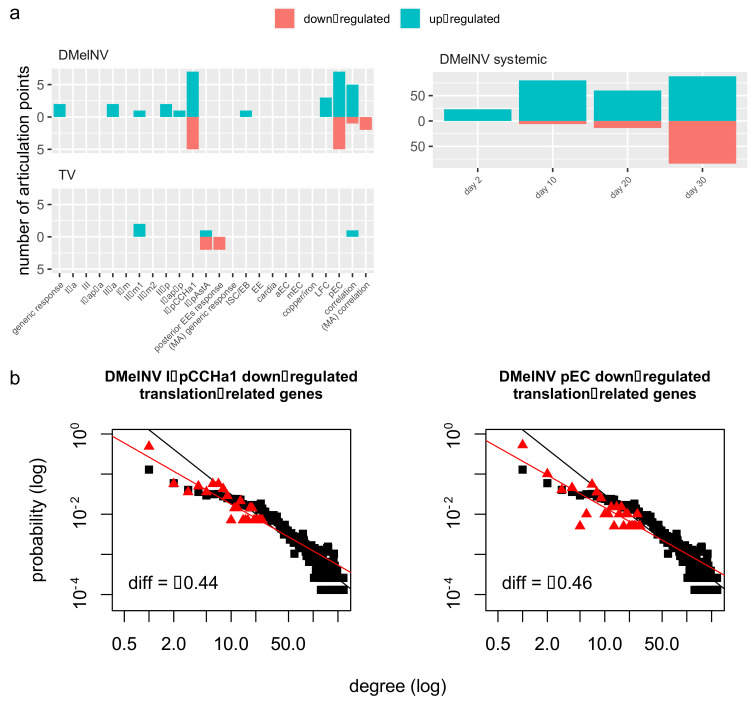
Perturbation of bottlenecks and hubs in the Drosophila interactome. (**a**) Number of articulation points found in each list of DEGs. (**b**) Down-regulated translation-related genes in response to DMelNV infection compose hubs in the drosophila interactome. Log-degree distributions of the whole *D. melanogaster* interactome (black) and subnetworks constructed with translation-related genes that are down-regulated in DMelNV-infected I-p^CCHa1^ and pEC cell subtypes (red). The slope of the regression lines represents the critical exponent of the power-law, γ.

While only a few DEGs in response to TV infection were found to constitute articulation points in the fly’s interactome, a higher disruption of articulation points was found in response to DMelNV infection (Figure 7a). In accordance, by comparing the betweenness centrality of DEGs to that of the whole interactome, a wide down-regulation of bottleneck genes in the systemic response to DMelNV infection was found in 20- and 30-day old flies (one-tailed Mann–Whitney U-test; adjusted *p* = 0.0027 and *p* < 0.0001, respectively). Given that bottlenecks bridge different parts of a graph, this result suggests that the flow of information to some components of the fly’s interactome is limited at later stages of infection in the systemic response to DMelNV.

Some genes related to heat shock response were found to comprise articulation points in the fly’s interactome, such as Hsc70-3 and Hsc70-4, Hsp83, and the MAPK-AK2 kinase, which suggests that heat shock response may play a role in activating antiviral defenses. Surprisingly, Hsc70-3 and Hsp83 are not up-regulated in the systemic response to DMelNV but rather down-regulated at later stages of infection (Figure 6c; Supplementary File S1).

Owning to the enrichment of translation-related genes that were down-regulated in the cell-type-specific response to DMelNV in the I-p^CCHa1^ EE and pECs subtypes, this subset of genes was mapped into the fruit fly’s interactome. As expected, given the essential role of translation, these subsets of genes represent hubs in the fly’s interactome (adjusted *p* = 0.0221 and *p* = 0.0493 for I-p^CCHa1^ EE and pEC, respectively; Figure 7b). In contrast, neither down-regulation of hub genes nor an enrichment of translational genes in down-regulated genes was found on the systemic response to DMelNV at any time point (Supplementary File S2).

### 3.6. Effects of Virus Infection on Cell Clustering and Cell-Type Annotation

**Figure 8:**
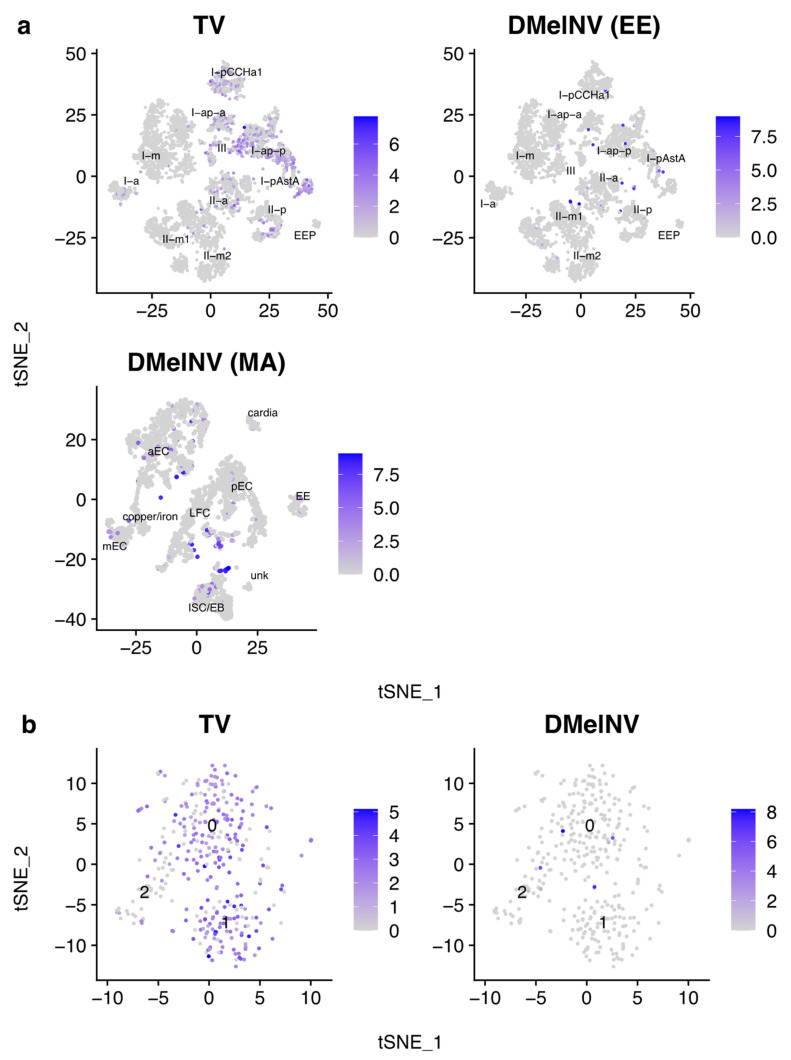
Confounding effects of viral infection on cell clustering and cell type annotation. t-SNE reduction plots of the (**a**) EE and MA datasets and (**b**) I-p^AstA^ EE subtype. Each point represents a single cell. The log(pseudocount + 1)-transformed proportion of viral counts (multiplied by a factor of 10,000) is shown for each cell.

We investigated whether virus infection can jeopardize cell clustering and cell-type annotation analyses given that unacknowledged viruses may be confounding factors in single-cell data. Cluster annotation on the EE and MA datasets appear to not be influenced by cell infection status although some infected cells seemed to be closer to each other on the t-SNE maps (Figure 8a). Overall, the EE and MA datasets are composed by heterogeneous sets of cells, and we asked whether the effects of infection might have a higher influence on clustering when analyzing a particular cell type/subtype. To investigate if this is the case, cells from the I-p^AstA^ subtype were subset, and we repeated the cluster generation analysis. Three clusters can be observed for the I-p^AstA^ subtype, of which cluster two appears to be composed mainly by uninfected cells (Figure 8b). These results indicate that 10^−4^ the effects of infection are more noticeable when analyzing more homogeneous group of cells.

## 4. Discussion

Cellular heterogeneity and complexity are masked by whole tissue and other population-averaged transcriptomic methods. These limitations can be overcome by single-cell technologies, which are able to measure the RNA expression profile of thousands of individual cells in a single batch [25]. Viral RNA kinetics in individual cells, especially in the case of polyadenylated viruses, can also be captured simultaneously with transcriptional changes to infection, making this a powerful tool for virologists. By analyzing scRNA-seq data from *D. melanogaster,* we showed that two picorna-like viruses, DMelNV and TV, employ different strategies regarding replication and alteration of host transcription on EEs. DMelNV was also found on a variety of cell types in a second scRNA-seq data of the whole fly midgut, and possibly, both DMelNV and TV infect other cell types not included in this study. Whereas DMelNV is known to be a non-pathological enteric virus, the biology of TV remains unknown, as this virus has been recently described by a metagenomic approach [14]. Here, we showed that TV’s capacity to infect and its replication level on EEs depended on the particular cell subtype being infected, and also, its accumulation was generally low compared to DMelNV (Figure 2). Up to 66% of the I-p^AstA^ subtype, located in the posterior region of the gastrointestinal tract, was infected with TV, while a low percentage of EEs from the gastric region was infected with this virus. On the other hand, DMelNV RNA accumulation was highly variable, sometimes exceeding 50% and up to nearly 80% of the total transcripts of a cell, and far less cells infected with this virus were detected on the EE dataset. Similar to DMelNV, a wide range in viral load/transcripts in infected cells was observed for other RNA viruses, such as vesicular stomatitis virus [49], dengue and zika viruses [50], poliovirus [51], foot-and-mouth disease virus [52], and influenza A virus (IAV) [8,53,54]. Cell subtype had a significant influence on viral RNA accumulation level. While TV accumulation was influenced by EE subtype, we failed to detect a significant influence of EE cell subtype on DMelNV accumulation and only detected a significant impact of cell type on its accumulation when analyzing cells from the entire midgut.

EEs response to TV and DMelNV infection was diverse, and different sets of genes were generally altered between TV- and DMelNV-infected cells. For TV, genes mostly related to glycosylation, insulin receptor recycling and insulin secretion, and cellular response to stress were enriched in cell-subtype-specific up-regulated genes lists in addition to pathways that suggest activation of the immune system (Figure 3b; Supplementary File S2). For DMelNV, genes mostly related to protein folding, cellular response to heat stress, and immune system (albeit distinct pathways to TV) were enriched in up-regulated genes, whereas genes related to translation and respiratory chain, among others, were enriched in down-regulated genes lists (Figure 3c; Supplementary File S2).

Viral RNA accumulation is expected to increase as a function of time; therefore, genes whose expression correlates to viral RNA accumulation may be indicatory of time of infection. Pathway enrichment analysis results for genes whose expression correlated to DMelNV accumulation were similar to the results obtained from DEGs lists, suggesting that overall, genes that respond to virus infection can also be used to estimate time of infection.

Genes associated with heat shock response were differentially expressed in both DMelNV- and TV-infected cells and the late systemic response to DMelNV. A previous study found that Hsp23, Hsp26, and Hsp70 were up-regulated in S2 cells challenged with DCV or invertebrate iridescent virus 6 (IIV-6), whereas only Hsp23 was up-regulated in response to cricket paralysis virus (CrPV). However, in whole adult flies, only Hsp23 and Hsp70 were up-regulated upon infection with DCV, and no up-regulation of heat shock proteins were detected upon IIV-6 infection. In contrast, Hsp70 was found to be up-regulated in adult flies infected with CrPV in addition to Hsp23 [13]. These results suggest differences between cellular and systemic heat shock response to viral infections and also specificity to particular viruses. Similarly, while we found Hsc70-3 and Hsc70-4 to be up-regulated as a generic response of EEs to DMelNV, and several heat shock proteins, such as Hsc70Cb, Hsp23, and Hsp83, were found to be up-regulated in a cell-subtype-specific manner, no similar up-regulation of heat shock proteins was found in the systemic response to this virus. Surprisingly, Hsc70-3, Hsc70Cb, and Hsp83 were down-regulated at 20 and 30 days post-eclosion in the systemic response to DMelNV.

Down-regulation of genes related to heat shock response was also observed in the cellular response to DMelNV and TV and might be attributed to viral mechanisms to suppress antiviral defenses. The elongation factor eEF1a1 was down-regulated in DMelNV-infected cells from the II-p EE subtype and in pECs and LFCs. Hsp26, 68, and 83 were down-regulated in TV-infected cells, whereas Hsp26 and 63 were down-regulated only in the I-p^AstA^ EE subtype (Figure 4).

Hsf regulon activity was higher in EEs and variable both between and within its subtypes (Figure 5). Given that overexpression of Hsf in transgenic fruit flies was accompanied by restriction of viral replication, sometimes even to undetectable levels [13], our results suggests that some EEs may have higher intrinsic immunity to viral infections.

Two cell subtypes showed a down-regulation of various proteins related to translation in response to DMelNV infection (Figure 3c; Supplementary File S2). Analysis of this subset of genes showed that they compose hubs in the fly’s interactome (Figure 4). This indicates a widespread shutoff of the translational machinery in these cell subtypes, possibly to tamper DMelNV replication. Alternatively, DMelNV targets only specific ribosomal genes as an attempt to suppress the translation of host RNA while enhancing the translation of its own proteins and therefore enhancing its own replication. Dysregulation of the translational machinery is a core function of ISGs in mammals [1,55], which corroborates with the first hypothesis. Previous microarray and bulk RNA-seq studies were unable to find a similar down-regulation of translation-related genes in the systemic response of DMelNV-infected flies [39,56].

Expression of bottleneck genes in the systemic response to DMelNV decreases at late stages of infection as the expression of immune genes increases (Supplementary File S2), suggesting a shift to a streamlined antiviral state in which resources are directed to the immune response. Further studies are necessary to test whether a late systemic down-regulation of bottlenecks in response to viral infections is a conserved phenomenon in drosophila and other models. Both RNA and DNA viruses were shown to target hubs and bottlenecks in their host’s interactome to regulate a wide range of cellular processes [57,58,59]. While we were not able to measure any direct protein–protein interaction in our analysis, a perturbation of both hubs and bottleneck genes in DMelNV-infected flies was found. According to the centrality-lethality rule, disruption of essential genes in the interactome might disentangle and dismantle it [48,60], which, on the one hand, might favor the virus infection process, while, on the other hand, it might diminish it. The down-regulation of translation-related genes in two cell subtypes infected with DMelNV and the down-regulation of bottleneck genes in the late systemic response to this virus are, more likely, related to antiviral response.

The heat stress-associated proteins Hsc70-3, Hsp83, and MAP-AK2 constitute articulation points in the fly’s interactome, suggesting they may play a role in activating antiviral pathways. If this is the case, it would be interesting to investigate whether a systemic antiviral response can be induced by cellular heat shock response. In contrast, we failed to detect any perturbation of hub genes in TV-infected cells, and a small number of articulation points were differentially expressed. As the time of infection is an important factor in regulation of the host transcriptome, and the scRNA-seq data used here are from 5–7 days old flies, we may have failed to detect a more substantial change to the host transcriptome at the cellular level. Given that this virus exhibited low RNA accumulation and elicited only moderate transcriptional response on infected cells where no significant modulation of essential genes was found, it is tantalizing to hypothesize that EEs are secondary targets of this virus.

We must acknowledge several drawbacks in our study. In addition to DMelNV, TV, and DCV, we also detected a non-polyadenylated virus, DAV, in the MA dataset in addition to the endosymbiontic bacteria *W. pipientis* in the EE dataset. The inability to precisely determine cells infected by non-polyadenylated viruses and intracellular bacteria means that some analyses, in particular differential expression, could be confounded by the presence of these pathogens. We also show that intrinsic heat shock response is highly variable, but the reasons for this variability is unknown. While some differences in the heat shock response between the EE and MA datasets might also be attributed to differences in the genetic background of the fruit fly or environmental factors, the heterogeneity in the cellular response to viral infections is already observed even within these datasets. Differences in the genetic background and environmental factors could also explain some differences between the cellular and systemic response to DMelNV, especially the lack of an up-regulation of the heat shock genes in the systemic response. Regardless of the motives, we show that heat shock response to viral infections is highly heterogeneous and cell-subtype- and virus-specific.

Lastly, given the presence of unacknowledged viruses in public scRNA-seq data, we hypothesize that viruses may be confounding factor in these kinds of experiments. In the datasets analyzed here, cells did not seem to cluster based on infection status when analyzing the EE and MA datasets as a whole (Figure 8a). However, when analyzing solely the I-p^AstA^ subtype, the effects of virus infection on cell clustering became more apparent (Figure 8b). Additionally, we have no information of which uninfected cells are responding to virus infections as bystanders, adding more hidden confounding factor to these analyses. One possibility to mitigate this problem would be to remove from the analysis all immune-related genes or genes that are correlated to viral load. A similar approach, where genes correlated to the top two principal components (PCs) composed by antiviral and inflammatory genes were omitted from the analysis, was performed to cluster pulmonary cells from IAV-infected and uninfected mice without the possible confounding effects of antiviral genes [8].

## 5. Conclusions

In this work, through analysis of in-vivo scRNA-seq data, we show the similarities and differences in the replication and infection strategies of two *D. melanogaster* viruses. We found a drastic contrast in the replication pattern between these two viruses. On the one hand, DMelNV only infected a few cells but exhibited high expression levels that sometimes exceeded 50% of the total mRNA of the cell, while, on the other hand, TV was able to infect a higher number of cells, but its replication level was generally low. Cells infected with either DMelNV or TV exhibited both generic and cell-subtype-specific transcriptional responses, and most importantly, heat shock response to viral infection was cell-subtype- and virus-specific. We detected a wide down-regulation of translation related genes in two cell subtypes in response to DMelNV infection. Further inspection of these translation-related genes revealed that they compose hubs in the host’s interactome. By analyzing publicly available bulk RNA-seq data from DMelNV-infected flies [39], we found that in this systemic response to DMelNV, bottleneck genes were down-regulated at 20 and 30 days post-eclosion. In contrast, no significant perturbation of hubs nor bottleneck genes were detected for TV.

## Figures and Tables

**Table 1:** DIAMOND BLASTX results. Hits to *Drosophila* and synthetic constructs were omitted.

Dataset	Contig	Contig Length	*E*-Value	Lowest Taxonomic Rank
EE	k99_30	813	7.1 × 10^−156^	Thika virus
	k99_1	541	1.2 × 10^−95^	*Saccharomyces cerevisiae*
	k99_22	402	9.5 × 10^−61^	*Saccharomyces cerevisiae*
	k99_3	306	1.8 × 10^−51^	Thika virus
	k99_24	543	2.2 × 10^−84^	Thika virus
	k99_7	328	2.0 × 10^−37^	*Muntiacus muntjak*
	k99_10	2627	0	Thika virus
	k99_9	490	1.5 × 10^−81^	Thika virus
	k99_28	319	1.1 × 10^−24^	*Wolbachia pipientis*
	k99_29	488	2.2 × 10^−56^	*Saccharomyces cerevisiae* YJM1401
	k99_14	349	4.8 × 10^−61^	Thika virus
	k99_16	1027	1.2 × 10^−189^	Thika virus
	k99_17	1694	0	Thika virus
	k99_39	12,357	0	Nora virus
	k99_21	337	3.0 × 10^−52^	Drosophila C virus
	k99_12	656	2.2 × 10^−54^	Drosophila C virus
	k99_36	3353	0	Drosophila C virus
	k99_38	551	1.3 × 10^−97^	Drosophila C virus
MA	k59_25	207	6.5 × 10^−13^	Nora virus
	k59_27	228	1.7 × 10^−19^	*Muntiacus reevesi*
	k59_45	984	1.2 × 10^−144^	Nora virus
	k59_62	2013	0	Nora virus
	k59_64	328	1.9 × 10^−43^	*Saccharomyces cerevisiae*
	k59_35	485	3.4 × 10^−41^	*Staphylococcus aureus*
	k59_72	754	3.8 × 10^−127^	Nora virus
	k59_77	281	5.3 × 10^−26^	*Macaca mulatta* polyomavirus 1
	k59_54	7298	0	Nora virus
	k59_58	344	4.6 × 10^−16^	Nora virus
	k59_29	313	1.3 × 10^−46^	*Drosophila melanogaster* tetravirus SW-2009a
	k59_21	443	4.2 × 10^−78^	Drosophila A virus
	k59_44	325	2.2 × 10^−52^	Drosophila A virus
	k59_32	328	1.2 × 10^−53^	Drosophila A virus
	k59_11	700	1.3 × 10^−86^	Drosophila A virus
	k59_36	414	6.3 × 10^−68^	Drosophila A virus
	k59_76	854	1.9 × 10^−167^	Drosophila A virus

## Data Availability

All R scripts used in this work, together with assembled contigs and count matrices are available at https://github.com/jmfagundes/dmelscrna, last accessed 15 November 2021.

## Supplementary Materials

The following are available online at https://www.mdpi.com/article/10.3390/v13112284/s1, Figure S1: marker genes used to identify clusters on the (a) EE dataset and (b) midgut atlas dataset; Supplementary File S1: list of DEGs found in the EE and MA datasets; Supplementary File S2: results for all pathway enrichment analyses and network analysis; Supplementary File S3: marker regulons for each cell cluster previously identified.
