# Supplementary figures and images for: Heterogeneity in the Response of Different Subtypes of Drosophila melanogaster Midgut Cells to Viral Infections
Source: Viruses. 2021 Nov 15;13(11):2284. doi: 10.3390/v13112284 (PMC8623525; doi:10.3390/v13112284)

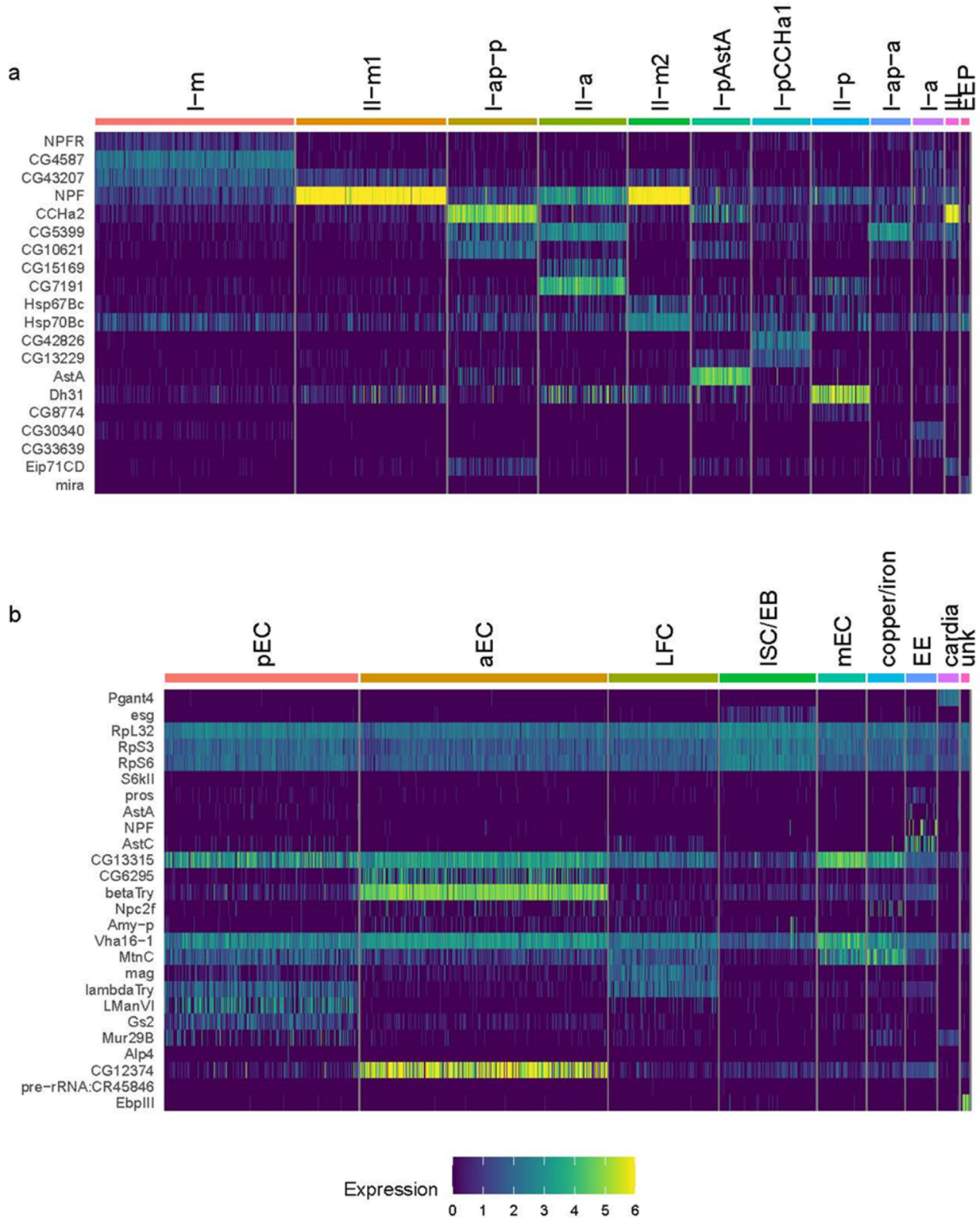

**Figure S1.** marker genes used to identify clusters on the (a) EE dataset and (b) midgut atlas dataset.

Supplement: Supplementary file 1 [file viruses-13-02284-s001.zip › Figure S1.pdf]
